# Immune Checkpoint Inhibitor Overlap Syndrome: Myocarditis, Myositis, and Myasthenia Gravis in a Patient With Metastatic Melanoma

**DOI:** 10.7759/cureus.105739

**Published:** 2026-03-23

**Authors:** Samuel Bennett, Abhinav Harish, Hamad Althumairy, Elisa Wang, Manu Mysore

**Affiliations:** 1 Internal Medicine, University of Maryland Medical Center, Baltimore, USA; 2 Pathology, University of Maryland Medical Center, Baltimore, USA; 3 Cardiology, University of Maryland Medical Center, Baltimore, USA

**Keywords:** immune checkpoint inhibitors, immune-related adverse events, ipilimumab, metastatic melanoma, myasthenia gravis, myocarditis, myositis, nivolumab, overlap syndrome

## Abstract

Immune checkpoint inhibitors can produce severe immune-mediated toxicity involving multiple organ systems. A rare manifestation is the myocarditis, myositis, and myasthenia gravis (MMM) overlap syndrome, also referred to as triple M syndrome or immune-related MMM overlap syndrome, characterized by concurrent myocarditis, myositis, and myasthenia gravis and capable of rapid progression to life-threatening cardiac and neuromuscular complications.

A 78-year-old man with metastatic melanoma developed palpitations, profound fatigue, proximal muscle weakness, diplopia, and ptosis two weeks after initiation of nivolumab and ipilimumab. Initial evaluation revealed marked elevations in high-sensitivity troponin (11,399 ng/L), creatine kinase (6,046 U/L), and transaminases, with sinus tachycardia on electrocardiography and preserved systolic function on echocardiography. Intravenous methylprednisolone was started, and immunotherapy was discontinued; however, his biomarkers continued to rise, and his condition rapidly deteriorated after transferring to a tertiary cardiac intensive care unit. He developed a complete heart block requiring transvenous pacing and subsequently demonstrated severe biventricular systolic dysfunction on cardiac imaging. Coronary angiography showed no obstructive disease. Despite escalation to pulse-dose steroids, his neuromuscular weakness worsened, and he progressed to respiratory failure requiring mechanical ventilation, followed by malignant ventricular arrhythmias and hemodynamic collapse before additional therapies, including plasmapheresis, could be initiated. Care was transitioned to comfort measures, and death occurred shortly thereafter. Endomyocardial biopsy confirmed lymphocytic myocarditis with myocyte necrosis.

This case highlights that early neuromuscular symptoms accompanied by marked troponin and creatine kinase elevation may represent the first manifestations of MMM overlap syndrome and can precede rapid progression to life-threatening cardiac and respiratory complications.

## Introduction

Immune checkpoint inhibitors (ICIs) are among the most transformative therapies in modern oncology and have demonstrated substantial survival benefits across a variety of malignancies, including melanoma, lung cancer, renal cell carcinoma, and Hodgkin lymphoma. These agents are used alone or in combination with chemotherapy and radiation, depending on cancer type [1]. ICIs function by targeting cytotoxic T-lymphocyte-associated antigen-4 (CTLA-4) or programmed cell death-1 (PD-1)/programmed death-ligand 1 (PD-L1), thereby removing inhibitory signals on T-cell activation and enabling a more robust antitumor immune response. Combination blockade of CTLA-4 and PD-1 produces broader immune activation but has been associated with higher rates of immune-related adverse events (irAEs) than with monotherapy [1].

Despite their clinical benefits, ICIs can lead to irAEs affecting nearly any organ system. The most common irAEs include colitis, hepatitis, pneumonitis, thyroiditis, and dermatitis [2]. Immune-mediated cardiotoxicity is uncommon but clinically significant, with presentations ranging from asymptomatic troponin elevation to sudden cardiac death [3]. Early reports described fulminant myocarditis occurring shortly after combination nivolumab and ipilimumab therapy, including fatal cases first reported by Johnson et al. [4]. Myocarditis remains the most fatal ICI-related cardiotoxicity, and when it occurs as part of overlap toxicity, the prognosis is particularly poor, with reported mortality ranging from approximately 38% to 60% in recent cohort and meta-analytic data [5,6]. In rare instances, patients develop a triad of myocarditis, myositis, and myasthenia gravis (MMM), referred to as “MMM” overlap syndrome, which has been estimated to occur in approximately 0.08% of treated individuals [5]. Myocarditis and myositis frequently co-occur, and myasthenia gravis has been reported in approximately 10% of myocarditis cases [7]. Because this syndrome is rare and reported predominantly in case reports and small series, early recognition can be challenging and may require a high index of suspicion outside oncology and cardiology subspecialty practice [3,8,9]. A 2024 systematic review of 50 cases found that the median time of onset after ICI therapy initiation was 21 days, highlighting the tendency for overlap toxicity to occur early after treatment initiation [10]. We present a case of biopsy-confirmed fulminant MMM overlap syndrome occurring within two weeks of combination nivolumab and ipilimumab therapy in metastatic melanoma. This case illustrates how early ocular and proximal neuromuscular symptoms accompanied by marked troponin and creatine kinase (CK) elevation may herald overlap toxicity and rapidly progress to complete heart block, cardiogenic shock, respiratory failure, and malignant ventricular arrhythmia.

## Case presentation

**Figure 1:**
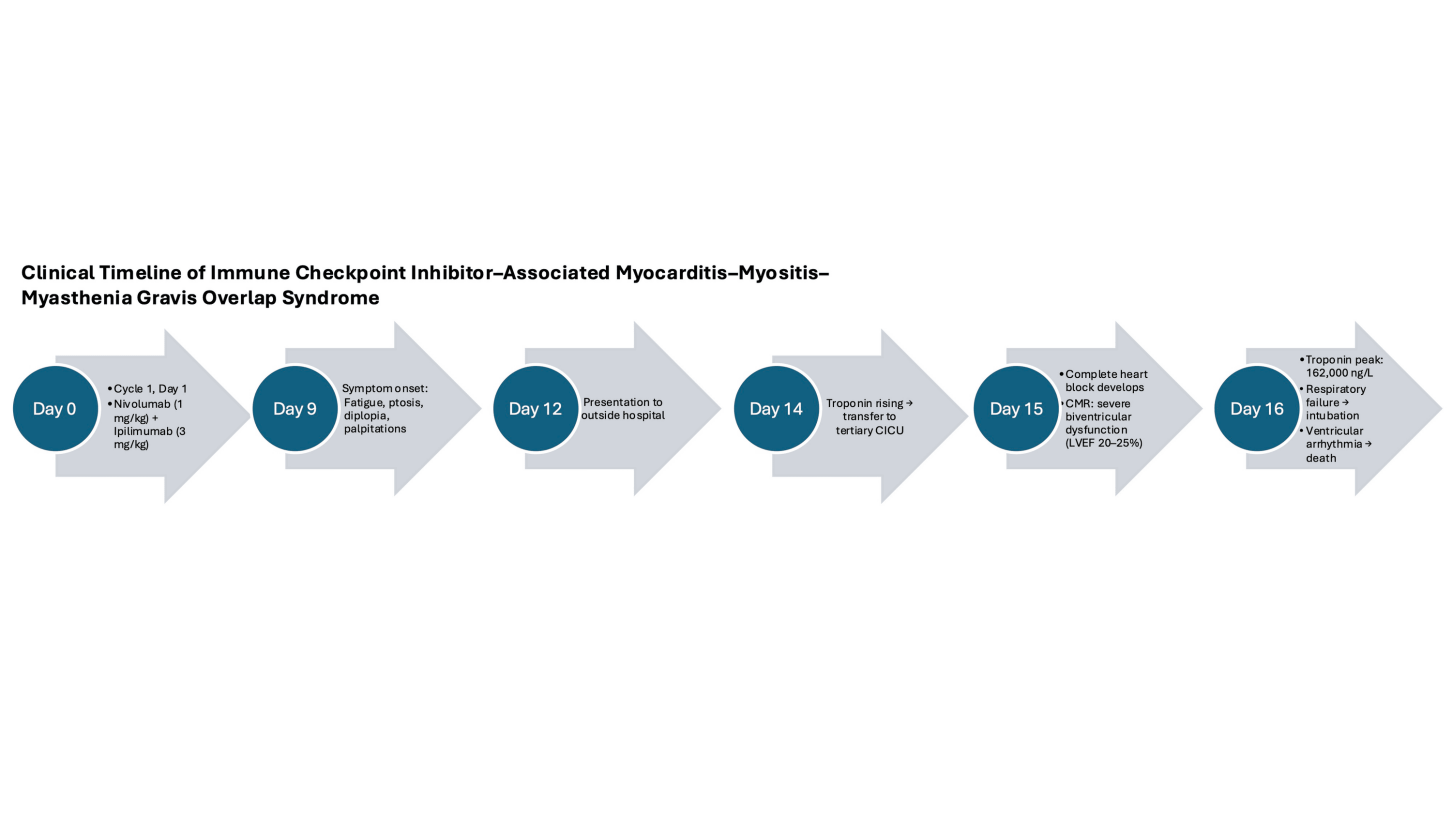
Clinical timeline beginning with cycle 1 day 1 of nivolumab/ipilimumab therapy and illustrating symptom onset, hospitalization, cardiac deterioration, and death in myocarditis, myositis, and myasthenia gravis overlap syndrome. CICU, cardiac intensive care unit; CMR, cardiac magnetic resonance imaging; LVEF, left ventricular ejection fraction.

A 78-year-old man with a history of hypertension, hyperlipidemia, and metastatic melanoma presented to an outside hospital with several days of palpitations, progressive fatigue, proximal muscle weakness, and intermittent diplopia and ptosis. He denied chest pain, dyspnea, or orthopnea. There was no known history of cardiomyopathy or prior structural heart disease. He had initiated cycle 1 day 1 of combination nivolumab (1 mg/kg) and ipilimumab (3 mg/kg) therapy 12 days before presentation. A timeline of the patient’s clinical course from immunotherapy initiation to death is shown in Figure 1.

On presentation, he was afebrile, with a blood pressure of 159/86 mmHg, a heart rate of 110 beats/min, and an oxygen saturation of 98% on room air. Physical examination demonstrated normal heart sounds, clear lung fields, and no peripheral edema. Laboratory evaluation revealed aspartate aminotransferase of 290 U/L (normal <34), alanine aminotransferase of 95 U/L (normal <49), troponin of 11,399 ng/L (normal <20), C-reactive protein of 28.8 mg/L (normal <10), and CK of 6,046 U/L (normal <294) (Table 1). The combination of marked troponin and CK elevation with ocular and proximal muscle weakness raised early concern for ICI overlap syndrome. Initial electrocardiography (ECG) demonstrated sinus tachycardia without ischemic ST-segment changes or atrioventricular conduction abnormalities. Transthoracic echocardiography (TTE) performed at the outside hospital demonstrated preserved/hyperdynamic left ventricular systolic function (estimated left ventricular ejection fraction or LVEF ≥65%) with normal right ventricular function and no conclusive regional wall motion abnormality, although the study was limited by tachycardia and technical factors. A prior echocardiogram was reportedly normal before initiation of ICI therapy, although the records were not available for review.

**Table 1:** Key laboratory findings during hospital course Creatine kinase declined after presentation, and the initial value represented the peak. C-reactive protein was not serially measured. ALT, alanine aminotransferase; AST, aspartate aminotransferase.

Test	Presentation	Peak	Unit	Reference range
Troponin	11,399	162,000	ng/L	<20
Creatine kinase	6,046	6,046	U/L	<294
AST	290	1,166	U/L	<34
ALT	95	624	U/L	<49
C-reactive protein	28.8	–	mg/L	<10

Given concern for ICI-associated myocarditis, immunotherapy was discontinued, and intravenous methylprednisolone 250 mg daily was initiated. Despite two days of corticosteroid therapy, troponin continued to rise to 24,670 ng/L, and the patient was transferred to our tertiary cardiac intensive care unit on hospital day 3 (approximately day 14 after ICI initiation).

**Figure 2:**
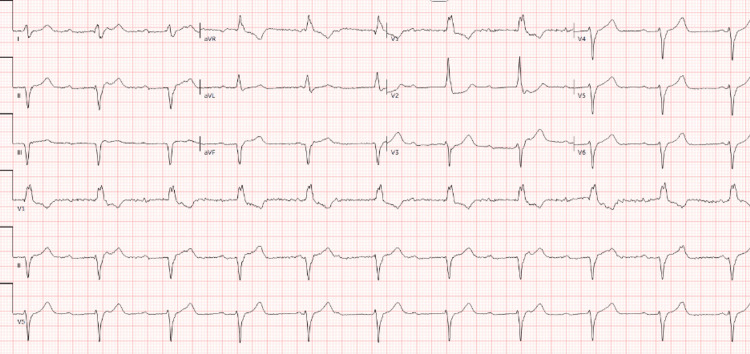
Electrocardiogram demonstrating complete heart block with junctional escape rhythm and a ventricular rate of approximately 40-60 beats/min.

**Figure 3:**
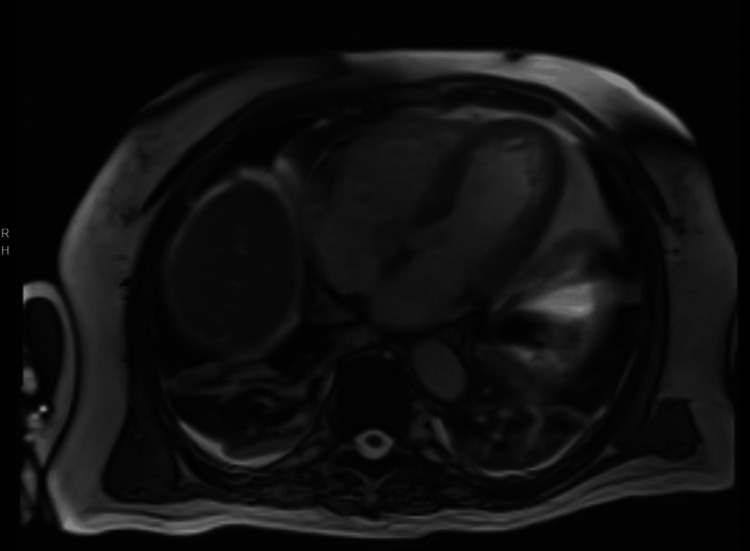
Cardiac magnetic resonance four-chamber view Cardiac magnetic resonance four-chamber view demonstrating severe biventricular systolic dysfunction (reported LVEF: 20%-25%). Tissue characterization was non-diagnostic due to motion artifact. LVEF, left ventricular ejection fraction.

**Figure 4:**
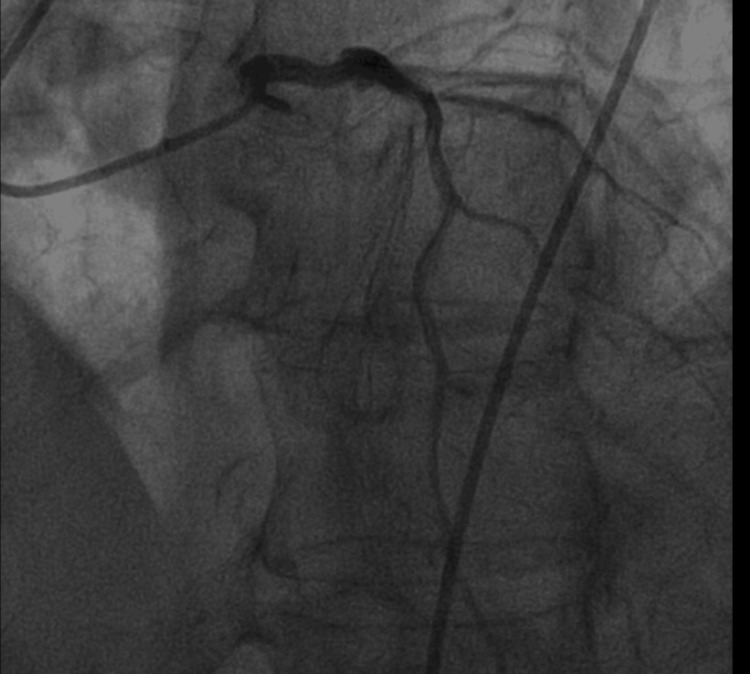
Coronary angiography demonstrating non-obstructive coronary artery disease.

Following transfer, ECG demonstrated complete heart block with junctional escape requiring transvenous pacing (Figure 2). Cardiac magnetic resonance imaging (CMR) showed severely reduced biventricular systolic function (LVEF: 20%-25%) but was non-diagnostic for tissue characterization due to motion artifact (Figure 3). The patient subsequently developed progressive conduction abnormalities and hemodynamic instability. Corticosteroid therapy was escalated to pulse-dose methylprednisolone (1 g daily). Right heart catheterization demonstrated elevated intracardiac filling pressures (right atrial pressure 22 mmHg, right ventricular pressure 48/18 mmHg, pulmonary artery pressure 36/13 mmHg (mean 20), and pulmonary capillary wedge pressure 15 mmHg) with reduced Fick cardiac output of 2.75 L/min and cardiac index of 1.41 L/min/m², consistent with cardiogenic shock physiology. Coronary angiography demonstrated no obstructive coronary artery disease (Figure 4). Endomyocardial biopsy (EMB) was performed.

Biomarkers continued to rise throughout hospitalization, with peak high-sensitivity troponin reaching 162,000 ng/L on the day of death. The patient subsequently developed acute respiratory failure requiring intubation in preparation for planned plasmapheresis. Shortly thereafter, the patient developed a malignant ventricular tachyarrhythmia with hemodynamic instability. Despite attempted cardioversion, the arrhythmia persisted. After discussion with the patient’s family, resuscitative efforts were withdrawn, and the patient was transitioned to comfort-focused care. Death occurred on hospital day 4, approximately 16 days after initiation of ICI therapy.

**Figure 5:**
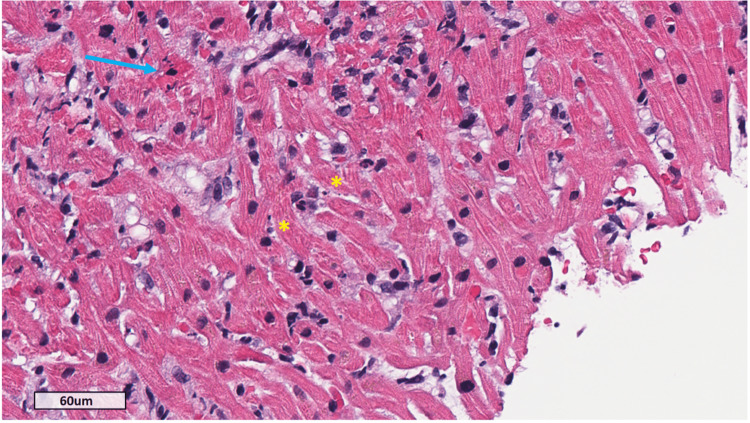
Endomyocardial biopsy Hematoxylin and eosin stain (original magnification ×40) demonstrating predominantly lymphocytic infiltrate with myocyte nuclear karyorrhexis and hypereosinophilia consistent with immune checkpoint inhibitor-associated myocarditis (blue arrow). Scattered apoptotic bodies are present (yellow asterisks). No giant cells were identified.

EMB confirmed lymphocytic myocarditis with myocyte necrosis consistent with ICI-associated myocarditis (Figure 5). 

## Discussion

This case illustrates the fulminant clinical course that can occur in MMM overlap syndrome following combination ICI therapy and highlights the diagnostic and management challenges associated with this rare toxicity. Although ICIs are generally well tolerated, fatal immune-related toxicity can occur. A meta-analysis of 112 trials involving 19,217 patients reported a toxicity-related fatality rate of 1.23% among those receiving combination PD-1/PD-L1 and CTLA-4 therapy. Fatal irAEs typically occurred early after therapy initiation, with a median onset of 14.5 days, and myocarditis carried the highest fatality rate [7]. Because these toxicities may present with non-specific multisystem symptoms, early recognition can be challenging outside oncology subspecialty practice and requires a high index of suspicion among clinicians caring for patients receiving ICIs.

Regarding the clinical presentation of each individual toxicity, proximal muscle weakness is the most common presenting feature of ICI-associated myositis, while ICI-associated myasthenia gravis (ICI-MG) may manifest with ptosis, diplopia, dysphagia, dysarthria, facial weakness, and fatigability. American Society for Clinical Oncology or ASCO guidelines recommend evaluation of CK, transaminases, and aldolase in suspected myositis [2]. Anti-acetylcholine receptor antibodies are detected in approximately two-thirds of ICI-MG cases [11]. Myasthenia gravis carries the risk of progressing to respiratory failure [2]. In this case, CK and transaminases were obtained at presentation and were markedly elevated, supporting concomitant myositis; however, aldolase and acetylcholine receptor antibody testing were not available before the patient’s rapid clinical deterioration.

ICI-associated myocarditis may present with chest pain, dyspnea, palpitations, fatigue, conduction abnormalities, and malignant arrhythmias. Cardiac biomarkers, including troponin and B-type natriuretic peptide (BNP), are frequently elevated and provide both diagnostic and prognostic value. In this case, BNP was not obtained during the patient’s initial evaluation. ECG abnormalities such as atrioventricular block and ventricular arrhythmia are common but non-specific. In this patient, the temporal relationship to recent ICI initiation, together with markedly elevated cardiac biomarkers and biopsy-proven lymphocytic myocarditis, strongly supported ICI-associated myocarditis. Alternative causes of atrioventricular block such as infiltrative cardiomyopathy, cardiac metastasis, or medication-related conduction disease were considered less likely. TTE is useful for initial evaluation and monitoring, while CMR is the most validated non-invasive diagnostic modality. EMB remains the diagnostic gold standard [12,13].

For management, high-dose corticosteroids remain first-line therapy. Current guidelines recommend pulse-dose methylprednisolone (1000 mg daily) for severe ICI-associated myocarditis. In this case, methylprednisolone 250 mg daily was initiated at the outside hospital before transfer and subsequently escalated to pulse-dose therapy after clinical deterioration. Adjunctive immunosuppressive therapies, including intravenous immunoglobulin G, rituximab, and plasmapheresis, have demonstrated variable success [10]. Guideline-directed therapies for ICI-MG may include pyridostigmine for symptomatic management and intravenous immunoglobulin or plasmapheresis in severe disease [2]. Increasing evidence supports the use of abatacept, including in combination with ruxolitinib, for steroid-refractory ICI-associated myocarditis; the largest cohort study to date reported substantially lower mortality among patients treated with abatacept-based therapy [14,15]. Robust evidence-based treatment algorithms for MMM overlap syndrome have not yet been established. Rapid clinical deterioration in this case limited the opportunity to initiate additional immunosuppressive therapies such as abatacept or ruxolitinib before hemodynamic collapse.

## Conclusions

MMM overlap syndrome is rare but associated with substantial morbidity and mortality. Specifically, it can cause severe complications, including respiratory failure, conduction abnormalities, and cardiogenic shock. New cardiopulmonary or neuromuscular symptoms occurring within weeks of ICI initiation, particularly when accompanied by marked troponin and CK elevation, should prompt urgent evaluation for overlap syndrome. Early recognition, discontinuation of ICI therapy, and prompt escalation of immunosuppressive treatment may be critical to limiting disease progression. As the diagnosis may not be immediately apparent at initial presentation, recognizing this constellation of findings may represent the only opportunity to intervene before rapid and irreversible clinical deterioration occurs.
